# Erythromycin, retapamulin, pyridoxine, folic acid, and ivermectin inhibit cytopathic effect, papain-like protease, and M^PRO^ enzymes of SARS-CoV-2

**DOI:** 10.3389/fcimb.2023.1273982

**Published:** 2023-11-27

**Authors:** Shaibu Oricha Bello, Mustapha Umar Imam, Muhammad Bashir Bello, Abdulmajeed Yunusa, Adamu Ahmed Adamu, Abdulmalik Shuaibu, Ehimario Uche Igumbor, Zaiyad Garba Habib, Mustapha Ayodele Popoola, Chinwe Lucia Ochu, Aishatu Yahaya Bello, Yusuf Yahaya Deeni, Ifeoma Okoye

**Affiliations:** ^1^ Department of Pharmacology and Therapeutics, Faculty of Basic Clinical Sciences, College of Health Sciences, Usmanu Danfodiyo University, Sokoto, Nigeria; ^2^ Nigerian COVID-19 Research Coalition, Nigerian Institute of Medical Research Institute, Lagos, Nigeria; ^3^ Centre for Advanced Medical Research and Training, College of Health Sciences, Usmanu Danfodiyo University, Sokoto, Nigeria; ^4^ Department of Medical Biochemistry, College of Health Sciences, Usmanu Danfodiyo University, Sokoto, Nigeria; ^5^ Department of Veterinary Microbiology, Faculty of Veterinary Medicine, Usmanu Danfodiyo University, Sokoto, Nigeria; ^6^ School of Public Health, University of the Western Cape, Cape Town, South Africa; ^7^ Department of Medicine, University of Abuja Teaching Hospital, Abuja, Nigeria; ^8^ Nigerian Centre for Disease Control and Prevention, Abuja, Nigeria; ^9^ Department of Clinical Pharmacy and Pharmacy Practice, Faculty of Pharmaceutical Sciences, Usmanu Danfodiyo University, Sokoto, Nigeria; ^10^ Department of Microbiology and Biotechnology, Federal University of Dutse, Dutse, Nigeria; ^11^ Centre for Environmental and Public Health Research and Development, Kano, Nigeria; ^12^ University of Nigeria Centre for Clinical Trials, University of Nigeria Teaching Hospital, Enugu, Nigeria

**Keywords:** erythromycin, retapamulin, pyridoxine, folic acid, ivermectin, SARS-CoV-2, COVID-19

## Abstract

**Background:**

Although tremendous success has been achieved in the development and deployment of effective COVID-19 vaccines, developing effective therapeutics for the treatment of those who do come down with the disease has been with limited success. To repurpose existing drugs for COVID-19, we previously showed, qualitatively, that erythromycin, retapamulin, pyridoxine, folic acid, and ivermectin inhibit SARS-COV-2-induced cytopathic effect (CPE) in Vero cells.

**Aim:**

This study aimed to quantitatively explore the inhibition of SARS-CoV-2-induced CPE by erythromycin, retapamulin, pyridoxine, folic acid, and ivermectin and to determine the effect of these drugs on SARS-CoV-2 papain-like protease and 3CL protease (M^PRO^) enzymes.

**Methods:**

Neutral red (3-amino-7-dimethylamino-2-methyl-phenazine hydrochloride) cell viability assay was used to quantify CPE after infecting pre-treated Vero cells with clinical SARS-Cov-2 isolates. Furthermore, SensoLyte^®^ 520 SARS-CoV-2 papain-like protease and SensoLyte^®^ 520 SARS-CoV-2 M^PRO^ activity assay kits were used to evaluate the inhibitory activity of the drugs on the respective enzymes.

**Results:**

Erythromycin, retapamulin, pyridoxine, folic acid, and ivermectin dose-dependently inhibit SARS-CoV-2-induced CPE in Vero cells, with inhibitory concentration-50 (IC_50_) values of 3.27 µM, 4.23 µM, 9.29 µM, 3.19 µM, and 84.31 µM, respectively. Furthermore, erythromycin, retapamulin, pyridoxine, folic acid, and ivermectin dose-dependently inhibited SARS-CoV-2 papain-like protease with IC_50_ values of 0.94 µM, 0.88 µM, 1.14 µM, 1.07 µM, and 1.51 µM, respectively, and inhibited the main protease (M^PRO^) with IC_50_ values of 1.35 µM, 1.25 µM, 7.36 µM, 1.15 µM, and 2.44 µM, respectively.

**Conclusion:**

The IC_50_ for all the drugs, except ivermectin, was at the clinically achievable plasma concentration in humans, which supports a possible role for the drugs in the management of COVID-19. The lack of inhibition of CPE by ivermectin at clinical concentrations could be part of the explanation for its lack of effectiveness in clinical trials.

## 1 Introduction

A major lesson learned from the Spanish flu of 1918 is that pandemics can rapidly decimate a population (Shanks et al., 2018). The COVID-19 pandemic, which started in Wuhan, China, in December 2019, had resulted in an estimated excess mortality of 18.9 million by the end of December 2022 with excess mortality being higher in developing economies and persons >60 years old (Shang et al., 2022). Beyond vaccines, it is important to identify therapeutics for those who do get infected and develop the disease. Although the world has risen to the challenge and there are currently over 400 drug candidates for the treatment of COVID-19, most are based on new molecules that, if found effective, are not expected to be available in developing economies within 2 years due to market prioritization mostly imposed by limited initial production capacities (Wise, 2022). Meanwhile, only a few therapies have consistently been found effective in ameliorating the duration of infection or the severity of COVID-19 (Ceramella et al., 2022). Other than dexamethasone and related steroids, lopinavir-ritonavir (Paxlovid) and remdesivir are not available in most countries and are out of reach to most people due to cost (Robinson et al., 2022). A huge therapeutic gap in COVID-19, therefore, remains.

COVID-19 is predominantly asymptomatic (80% of cases) (de Sousa et al., 2020). When symptomatic, the clinical presentation is that of respiratory infection with severity ranging from a mild common cold-like illness to severe viral pneumonia leading to acute respiratory distress syndrome that is potentially fatal (de Sousa et al., 2020). Knowledge of the pathogenesis of COVID-19 is still evolving but the structure of the virus, mechanisms of viral entry, and replications are essentially settled and appear target rich for drug discovery and development. It is, therefore, difficult to explain candidate therapies that perform excellently *in vitro* but fail in clinical trials. The reasons may include the presence of unresolved redundant pathways or a dis-linkage between viremia and pathology. It is becoming clear that combination therapies (or single therapy) that target multiple pathways in the pathogenesis of COVID-19 may prove superior.

Our team previously developed and validated an algorithm that involves deliberate consideration of multiple targets, cost, toxicity, and availability in selecting drugs for rapid repurposing efforts with an off-label application as the immediate goal (Bello et al., 2023).

SARS-CoV-2-induced cytopathic effect (CPE) has previously been validated as a surrogate for SARS-CoV-2 infectivity, and it is non-inferior to PCR (Basile et al., 2021). Therefore, in our previous study (Bello et al., 2023), the demonstration of inhibition of CPE was considered more cost-effective and a surrogate of clinical efficacy than the demonstration of inhibition of viral multiplication alone due to the possibility of a threshold effect in viral-induced CPE (Shahsavandi et al., 2013). We also identified erythromycin, pyridoxine, folic acid, and ivermectin as potential drugs to repurpose for COVID-19; with saw a wet laboratory demonstration of inhibition (qualitative assay) of SARS-COV-2-induced CPE in Vero cells and *in silico* prediction of inhibition of multiple critical SARS-CoV-2 enzymes (Bello et al., 2023).

This study was conducted to further evaluate erythromycin, pyridoxine, folic acid, and ivermectin as potential drugs for COVID-19 by quantitatively measuring the inhibitions of CPE, SARS-CoV-2 papain-like protease, and SARS-CoV-2 3CL main protease (M^PRO^).

## 2 Materials and methods

### 2.1 Materials

SensoLyte® 520 SARS-CoV-2 M^PRO^ Activity Assay Kit, SensoLyte® 520 SARS-CoV-2 papain-like protease/Deubiq, molecular grade water, neutral red (3-amino-7-dimethylamino-2-methyl-phenazine hydrochloride) (Solarbio, cat. N8160), Minimal Essential Medium/Earls Balance Salts (MEM/EBSS) (HyClone Laboratories, Utah, USA), glacial acetic acid, ethanol 96% 0.4% (wt./vol), Trypan blue in 0.9% NaCl solution, SARS-CoV-2, virus (clinical isolate), and cell culture from Nest (Jiangsu, China) were used in this study.

### 2.2 Cell culture procedure

Vero cell preparation, passaging, SARS-CoV-2 virus, and sources of experimental drugs were as previously described (Bello et al., 2023). Three different doses of each drug were tested for antiviral activity: erythromycin, retapamulin, folic acid, and ivermectin were tested at 5 µM, 7.5 µM, and 10 µM, while pyridoxine was tested at 10 µM, 15 µM, and 20 µM, all in three independent replicates.

Briefly, we seeded 96-well plates with 6 × 10^4^ cells/mL of Vero E6 (200 μL per well), using Minimum Essential Medium (MEM) with 10% fetal bovine serum (FBS) without antibiotics. Plates were incubated overnight at 37°C in a 5% CO_2_ atmosphere. The following day, the 96-well plates were viewed under an inverted microscope for a confluence of approximately 50%.

Sixty minutes before drug treatment, cell culture supernatant was removed from each well and the wells were washed with 150 μL of phosphate-buffered saline (PBS). Except for the negative and cell growth control wells where 50 μL of PBS was added, each well was infected with 50 μL of SARS-CoV-2 diluted in PBS at a multiplicity of infection (MOI) of 0.1 and incubated for 1 h at 37°C in 5% CO_2_ with intermittent shaking of the plates at 15-min intervals to allow for viral adsorption. Thereafter, the infection supernatant was removed and 200 μL of the respective drugs diluted in MEM/EBSS having 1% FBS without antibiotics were added to the different treatment groups and incubated at 37°C in 5% CO_2_. The cells were viewed using an inverted microscope after 48 h to check for CPE.

### 2.3 Quantification of inhibition of SARS-COV-2-induced CPE using neutral red assay

Neutral red (NR) assay (Bello et al., 2023) was used to quantify cell viability and the inhibition of CPE. The NR uptake assay provides a quantitative estimation of the number of viable cells in the culture. It is based on the ability of viable cells to incorporate and bind the neutral red dye in the lysosomes, which are then extracted using glacial acid for measurement of optical densities using a spectrophotometer (Bello et al., 2023).

Briefly, an overnight-incubated 40 µg mL^−1^ NR working solution (in MEM) was filtered using a 2-µm membrane filter to remove any precipitated dye crystals. The attached cells from the *in vitro* antiviral activity experiments were washed with 150 μL of PBS per well and the washing solution was removed. The NR medium was gently placed in a reservoir and 100 μL of the NR medium was pipetted to each well of the plates. The plates were incubated for 2 h under the proper culture conditions. After the period of incubation, the plates were inspected with an inverted microscope to check the possible precipitation of NR. The medium was removed, and the cells were washed two times with 150 μL of PBS per well. Thereafter, 150 μL of NR destain solution was added per well and the plates were shaken rapidly on a microtiter plate shaker at 500 rpm for 10 min.

The optical densities of extracted neutral red were measured at 540 nm in a microtiter plate reader. Blank was subtracted from the resulting absorbance values before data analysis. The groups were (i) virus-infected cells (virus control), (ii) virus infected and treated cells (treatment group), and (iii) cells with no virus or drug treatment (growth control). Growth and inhibition of CPE were determined relative to the growth of the untreated control.

### 2.4 Inhibition of SARS-CoV-2 papain-like protease activity

All working solutions were prepared according to the manufacturer’s instructions.

The enzymatic reaction was set up by adding 10 μL per well of three respective concentrations of erythromycin, retapamulin, folic acid, and ivermectin (2.5 µM, 5 µM, and 10 µM) and pyridoxine (5 µM, 10 µM, and 20 µM). Dose spacing was designed to be within the achievable plasma concentration of the drugs as derived from the literature maximum serum concentrations (C_max_) at routine doses of the drugs. Each test was done in triplicate.

Briefly, 40 μL of diluted enzyme working solution was added to each microplate well. Simultaneously, the following control wells were set up.

i. Positive control containing 40 μL of papain-like protease without the test drugs.
ii. Inhibitor control containing 40 μL of papain-like protease and 10 μL of GRL0617 (manufacturer-supplied inhibitor).
iii. Vehicle control containing (a) 40 μL of papain-like protease and (b) 10 μL of 0.1% DMSO (vehicle used in delivering test compound).
iv. Test compound control containing assay buffer and 10 μL of test compound.
v. Substrate control containing assay buffer.

To each well, papain-like protease and substrate solution was added, and the reagents were mixed by shaking the plate gently for 30 s. The plates were then incubated away from direct light at 37°C for up to 60 min. The fluorescence intensity was measured at an excitation and emission wavelength of 490 nm and 520 nm, respectively.

### 2.5 Inhibition of SARS-CoV-2, M^PRO^ activity

The experimental setup and the relevant controls were as with the inhibition of SARS-CoV-2 papain-like protease activity assay, except the SARS-CoV-2 M^PRO^ enzyme and activity assay kit was used according to the manufacturer’s protocol. Sensolyte® Protocol A was used for screening 3CL protease (M^PRO^) inhibitors using purified enzyme. Briefly, to obtain the working solutions, 3CL protease enzyme was diluted 80-fold in assay buffer, while 3CL protease substrate (Component A) and inhibitor (GC 376) (positive control) were diluted 100-fold in assay buffer. The enzymatic reaction was set up as for papain-like proteases above except that GC376 was the inhibitor. The volume of enzyme and test compound for each well was 40 μL and 10 μL, respectively. Assay buffer was used to bring the total volume of all controls to 50 μL.

## 3 Data analysis

Statistical software GraphPad Prism 9.4.1, NCSS 2022, and Microsoft Excel were used for data analysis and graphical representations. We checked data for outliers using Tukey’s box-plot method (interquartile principle) (Mowbray et al., 2019). However, outliers were included in the analysis of CPE inhibition because biological reasons may be involved (Mowbray et al., 2019). Otherwise, we substituted outliers by the group average in the enzyme assays because precision is more likely involved (Zhang et al., 2020). Nonetheless, sensitivity analysis was conducted by including outliers in the enzyme assays. Percentage inhibition of CPE was calculated from the optical densities according to Severson et al. (2007): $\frac{\left(Test\;substance-Virus\;control\right)}{\left(Cell\;control-Virus\;control\right)}*100$ , provided the concentrations of test drug were non-toxic to Vero cells in a separate assay. Although Severson et al. considered inhibition of CPE ≥ 50% as hits in their study, we accepted inhibitions of CPE ≥ 15% as hits because this was not a full-range dose–response study, but concentrations were restricted to those achievable within the human plasma at routine dosing schedules. Maximum responses (efficacy) were, therefore, conceivably different from what we observed. Percentage inhibition of enzyme activity was calculated according to the manufacturer’s instruction and essentially represented as $\frac{\text{RFU Negative control }–\text{ RFU Test}}{\text{RFU Negative control }}*100$ (after subtracting the blank), where RFU is the relative fluorescent unit. The concentration that gave a 50% response (EC_50_ or IC_50_) was determined by first visual inspection of the dose–response plot to infer possible models (Sebaugh, 2011). Maximum and minimum responses were constrained to 100% and 0%, respectively, because these were the only possible outcomes in data normalized to percentage response. Nonlinear regression three-parameter dose–response models were used on the primary runs but compared to alternate models with higher R-square used to decide the best model. Furthermore, a 95% confidence interval (CI) was determined for all estimates. In determining the 95% CI, unknowns were interpolated from the standard curve. In the CPE evaluation, all comparisons were to SARS-CoV-2-infected but vehicle (DMSO)-treated Vero cells. In the enzyme inhibition assay, all comparisons were to the uninhibited enzyme activity. In both enzyme inhibition assays, manufacturer-supplied inhibitor controls were used to ascertain the integrity of the system. The level of significance was set at *p*< 0.05.

## 4 Results

### 4.1 Inhibition of SARS-CoV-2-induced CPE

All tested doses of the drugs alone were not toxic to Vero cells.

**Figure 1:**
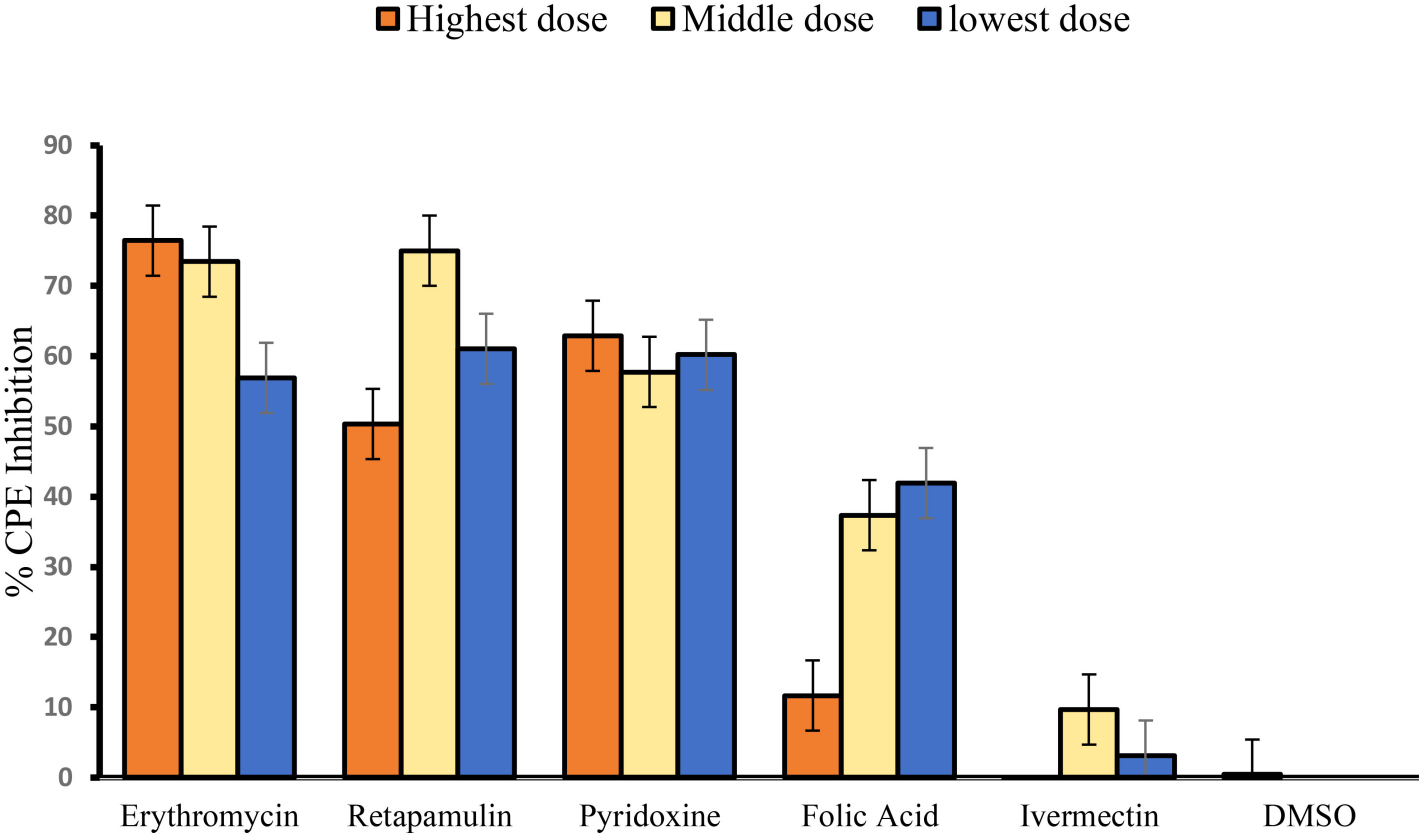
Inhibition of SARS-CoV-2-induced CPE. For erythromycin, retapamulin, folic acid, and ivermectin, high dose, medium dose, and low dose respectively represent 10 µM, 7.5 µM, and 5 µM, but for pyridoxine, the doses were 20 µM, 15 µM, and 10 µM. The doses were selected based on achievable C_max_ in human subjects at standard doses of the drugs. All concentrations of test drugs were found nontoxic to Vero cells. Analyses were performed with outliers because biological factors probably dictated differences (Mowbray et al., 2019).

Erythromycin, retapamulin, pyridoxine, and folic acid dose-dependently and significantly (*p* = 0.0005) inhibited SARS-CoV-2-induced CPE with a maximum inhibition of 76%, 75%, 63%, and 42% respectively at the tested doses. However, the maximum inhibition of CPE by ivermectin was 10% (at 10 µM) and this was not significant (*p* = 0.5) (**Table 1**; **Figure 1**).

**Table 1:** Inhibition of SARS-CoV-2-induced CPE in Vero cells.

Drug	Maximal inhibition of CPE (%)	Dose at maximal observed* inhibition of CPE (µM)	IC_50_ (µM)	95% CI of IC_50_ (µM)	*p*
Erythromycin	76	10	3.27	2.68 to 3.93	<0.05
Retapamulin	75	7.5	4.23	2.29 to 7.22	<0.05
Pyridoxine	63	20	9.29	6.30 to 13.31	<0.05
Folic Acid	42	5	3.19	2.16 to 4.49	<0.05
Ivermectin	10	7.5	84.31	41.70 to 759.2	>0.05

* “Maximal observed” inhibition in this study is not the inherent maximum possible inhibition efficacy of the tested drug, but that observed within the dose range tested. These dose ranges were centered at an achievable plasma concentration of the drugs at the currently recommended doses except ivermectin where ranges were derived from the literature of concentrations previously found to inhibit SARS-CoV-2 proliferation (Caly et al., 2020; Momekov and Momekova, 2020; Schmith et al., 2020). Ivermectin causes non-significant inhibition of CPE and then at an inhibitory concentration-50 (IC_50_) not achievable in human plasma (800 mcg/kg maximum single dose of ivermectin has a C_max_ of 108.1 ng/mL or 0.124 µM/mL) (Smit et al., 2016). Analyses were performed with outliers because biological differences probably dictated differences (Mowbray et al., 2019).

### 4.2 Inhibition of SARS-CoV-2 papain-like protease

**Figure 2:**
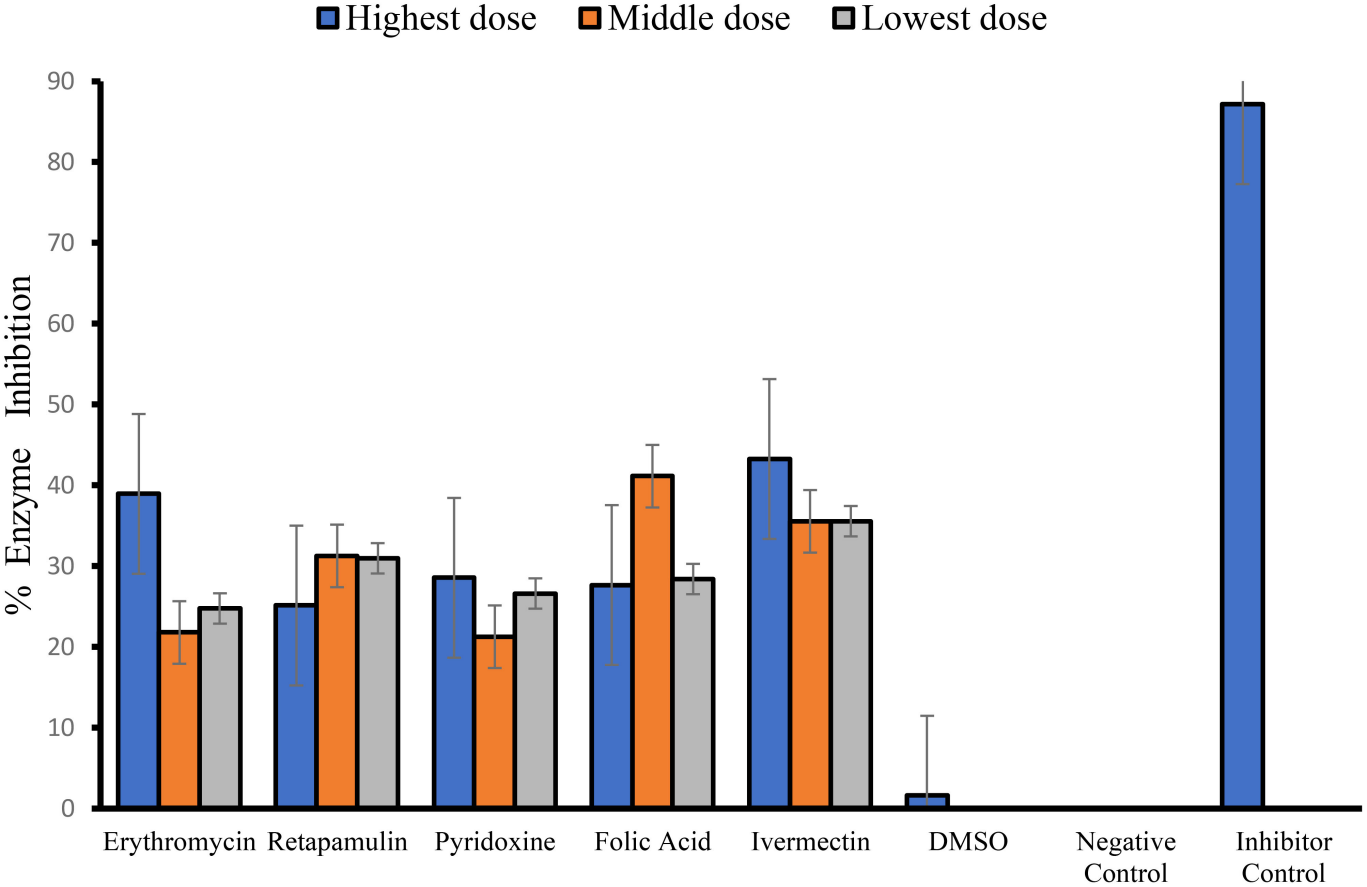
Inhibition of SARS-CoV-2 papain-like protease. For erythromycin, retapamulin, folic acid, and ivermectin, high dose, medium dose, and low dose respectively represent 10 µM, 5 µM, and 2.5 µM, but for pyridoxine, the doses were 20 µM, 10 µM, and 5 µM. The doses were selected based on achievable C_max_ in human subjects at standard doses of the drugs and to input the dose doubling escalation method. All concentrations of test drugs were found nontoxic to Vero cells. Analyses were performed with outliers substituted by averaging or neighborhood method because precision rather than biological factors probably dictated differences (Mowbray et al., 2019).

Erythromycin, retapamulin, pyridoxine, folic acid, and ivermectin dose-dependently and significantly (*p*< 0.05) inhibited SARS-CoV-2 papain-like protease with a maximum inhibition of 29.46%, 20.60%, 17.46%, 31.99%, and 34.34%, respectively, at the tested doses (**Table 2**; **Figure 2**).

**Table 2:** Inhibition of SARS-CoV-2 papain-like protease (PP).

Drug	Maximal inhibition of PP (%)	Dose at maximal observed* inhibition of PP (µM)	IC_50_ (µM)	95% CI of IC_50_ (µM)	*p*
Erythromycin	29.46	10.0	0.94	0.20 to 2.09	<0.05
Retapamulin	20.60	5	0.88	0.45 to 1.42	<0.05
Pyridoxine	17.46	20.0	1.14	0.30 to 2.23	<0.05
Folic Acid	31.99	5	1.07	0.47 to 1.90	<0.05
Ivermectin	34.34	10	1.51	0.55 to 3.07	<0.05

*”Maximal observed” inhibition in this study is not the inherent maximum possible inhibition efficacy of the tested drug, but that observed within the dose range tested. These dose ranges were centered at an achievable plasma concentration of the drugs at the currently recommended doses except ivermectin where ranges were derived from the literature of concentrations previously found to inhibit SARS-CoV-2 proliferation (Caly et al., 2020; Momekov and Momekova, 2020; Schmith et al., 2020). Ivermectin causes significant inhibition of PP but at an IC_50_ concentration not achievable in human plasma (800 mcg/kg maximum single dose of ivermectin has a C_max_ of 108.1 ng/mL or 0.124 µM/mL) (Smit et al., 2016). Analyses were performed with the substitution of outliers because precision rather than biological factors probably dictated differences (Mowbray et al., 2019).

### 4.3 Inhibition of SARS-CoV-2 Main Protease (M^PRO^ or 3CL protease)

**Figure 3:**
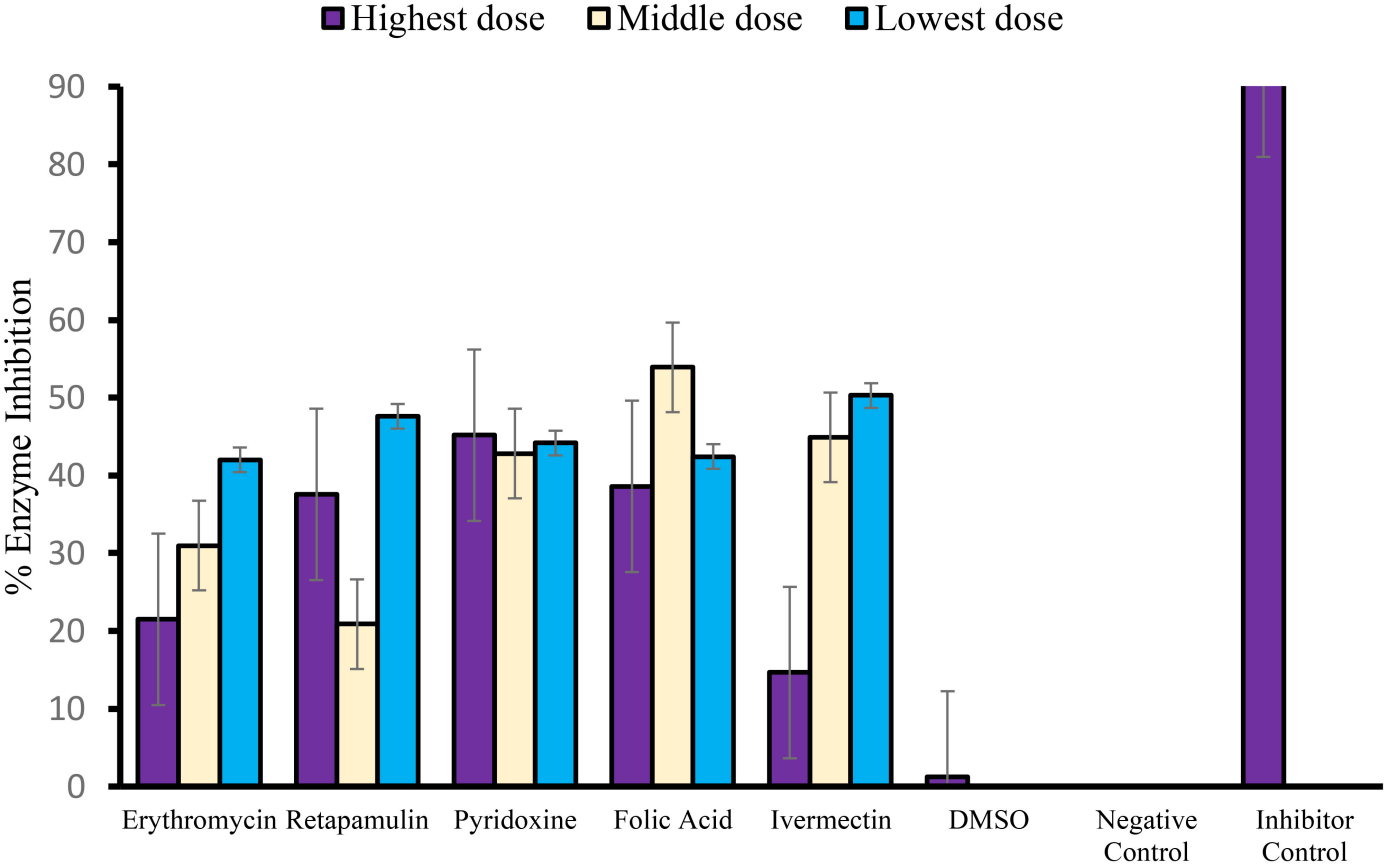
Inhibition of SARS-CoV-2 main protease (MPRO or 3CL protease). For erythromycin, retapamulin, folic acid, and ivermectin, high dose, medium dose, and low dose respectively represent 10 µM, 5 µM, and 2.5 µM, but for pyridoxine, the doses were 20 µM, 10 µM, and 5 µM. The doses were selected based on achievable C_max_ in human subjects at standard doses of the drugs and to input the dose doubling escalation method. All concentrations of test drugs were found nontoxic to Vero cells. Analyses were performed with outliers substituted by averaging or neighborhood method because precisions rather than biological factors probably dictated differences (Mowbray et al., 2019).

Erythromycin, retapamulin, pyridoxine, folic acid, and ivermectin dose-dependently and significantly (*p*< 0.05) inhibited SARS-CoV-2 M^PRO^ with a maximum inhibition of 30.51%, 33.37%, 45.1%, 26.8%, and 38.07%, respectively, at the tested doses (**Table 3**; **Figure 3**).

**Table 3:** Inhibition of SARS-CoV-2 main protease (MPRO or 3CL).

Drug	Maximal inhibition of M^PRO^ (%)	Dose at maximal observed* inhibition of M^PRO^ (µM)	IC_5 0_(µM)	95% CI of IC_50_ (µM)	*p*
Erythromycin	30.51	2.5	1.35	0.90 to 1.91	<0.05
Retapamulin	33.37	2.5	1.25	0.60 to 2.15	<0.05
Pyridoxine	45.17	20.0	7.36	4.14 to 12.67	<0.05
Folic Acid	26.8	2.5	1.15	0.81 to 1.56	<0.05
Ivermectin	38.07	5.0	2.44	1.49 to 3.77	<0.05

*”Maximal observed” inhibition in this study is not the inherent maximum possible inhibition efficacy of the tested drug, but that observed within the dose range tested. These dose ranges were centered at an achievable plasma concentration of the drugs at the currently recommended doses except ivermectin where ranges were derived from the literature of concentrations previously found to inhibit SARS-CoV-2 proliferation (Caly et al., 2020; Momekov and Momekova, 2020; Schmith et al., 2020). Ivermectin causes significant inhibition of M^PRO^ but at an IC_50_ concentration not achievable in human plasma (800 mcg/kg maximum single dose of ivermectin has a C_max_ of 108.1 ng/mL or 0.124 µM/mL) (Smit et al., 2016). Analyses were performed with the substitution of outliers because precision rather than biological factors probably dictated differences (Mowbray et al., 2019).

## 5 Discussion

This study was conducted as a standalone evaluation of the effect of drugs previously identified by *in silico* screening (Bello et al., 2023) as potential therapeutics for SARS-CoV-2 infection and COVID-19. Although the effect of the drugs on SARS-CoV-2-induced CPE in Vero cells, SARS-CoV-2 main protease (M^PRO^), and papain-like protease (PP) was evaluated, these were not considered necessarily linked (mechanistic) because the initial *in silico* selection was based on predicted activity at 11 independent targets (Bello et al., 2023), any combination of which may be related to observed inhibition of CPE. Interestingly, the nirmatrelvir–ritonavir combination (Paxlovid) (Ng et al., 2022; Yang et al., 2022), the current forerunner in COVID-19 therapeutics, is an M^PRO^ inhibitor and it is accepted that its efficacy is related to this inhibition (Pavan et al., 2021).

Erythromycin, retapamulin, and pyridoxine significantly inhibited SARS-CoV-2-induced CPE in Vero cells to an extent that met our criteria (based on anticipated combination therapy) (≥15%) and that of Severson et al. (based on anticipated monotherapy) (≥50%) (Severson et al., 2007) to define hits. It is reassuring that the CPE inhibitions were at concentrations and IC_50_ that were consistent with achievable plasma levels at currently recommended doses (erythromycin and pyridoxine) or formulations (retapamulin) of these drugs. Furthermore, erythromycin, retapamulin, and pyridoxine significantly inhibited SARS-CoV-2 M^PRO^ and PP at achievable concentrations and IC_50_. To the best of our knowledge, this is the first study to identify erythromycin and retapamulin as potential therapeutics for COVID-19. Pyridoxine has previously been reported as a useful supplementary therapy for COVID-19, but this was in a context unrelated to its antiviral activity (Beigmohammadi et al., 2020; Ibrahim et al., 2022).

Folic acid significantly inhibited SARS-CoV-2-induced CPE with a maximum inhibition of 42% at the clinically achievable concentration of 5 µM, thus satisfying our criteria for hits but falling short of the criteria of Severson et al. (2007). Furthermore, the dose–response pattern suggests that lower doses are more effective than higher doses, suggesting that higher doses may be toxic to Vero cells in the presence of SARS-CoV-2 viruses because, as reported above, the tested doses of the drug (alone) were not found to be toxic to Vero cells in the cell toxicity study. This pattern of the lower dose being more effective is also observed in folic acid’s significant inhibition of SARS-CoV-2 M^PRO^. The immediate implication of these findings is uncertain beyond the desirable recommendation that lower doses of folic acid should be the target of future possible utilization of folic acid in SARS-CoV-2 infections. This is contrary to the suggestion of Asad and Shuja (2021) that a higher dose of folic acid would be beneficial in COVID-19, though the suggestion was based on studies that examined the inhibition of Spike protein by folic acid (Abd El Hadi et al., 2021). Folic acid also significantly inhibits SARS-CoV-2 papain-like protease at concentrations and IC_50_ that is also achievable at routine therapeutic doses. Indeed, previous molecular docking studies by our group (Bello et al., 2023) and others (Lokhande et al., 2021) suggest folic acid as a potential therapeutic in COVID-19. Wet laboratory studies further suggest that folic acid inhibits SARS-COV-2 nucleocapsid protein (Chen et al., 2022) and inhibits cell invasion by SARS-CoV-2 by methylating ACE2 (Zhang et al., 2022). The relationship between enzyme inhibition and inhibition of CPE is therefore not immediately certain. Perhaps, a combination effect may be an explanation.

Ivermectin significantly inhibited SARS-CoV-2 M^PRO^ and PP with either greater or equivalent percentage inhibition compared to other drugs tested. Nonetheless, ivermectin failed to significantly inhibit SARS-CoV-2-induced CPE. These findings may partly explain the lack of clinical efficacy of ivermectin in various clinical trials (López-Medina et al., 2021; Popp et al., 2021; Lim et al., 2022) irrespective of the findings in other studies that, at very high doses (1,200 µg/kg), started early in the disease (Buonfrate et al., 2022), ivermectin potently inhibits viral replication with up to 5,000- fold reduction in viral load (Caly et al., 2020). In any case, it is important to note that the concentrations of ivermectin under consideration (2.5–5 µM) are not achievable clinically at currently recommended doses of<200 µg/kg (Chaccour et al., 2020; Momekov and Momekova, 2020; Schmith et al., 2020). Nonetheless, our findings further point towards CPE and/or cell-based assays as, perhaps, more clinically relevant endpoints in screening therapeutics against SARS-CoV-2 (Severson et al., 2007; Basile et al., 2021; Ngan et al., 2022).

The antiviral effects of erythromycin, retapamulin, pyridoxine, folic acid, and ivermectin elicited in this study are at least partly related to the inhibitions of SARS-COV-2 protease enzymes (3CL and papain proteases), thereby preventing procession of viral polyprotein, viral protein maturation, and packaging. Similar antiviral mechanisms (prevention of viral protein processing) have been established in antiretroviral agents (Mahdi et al., 2020) and have been identified in anti-SARS-COV-2 drug under development (Iketani et al., 2021). In this regard, it is interesting that the currently approved combination drugs for SARS-COV-2 infection, nirmatrelvir/ritonavir (Paxlovid), contain inhibitors of SARS-CoV-2 main protease (Nirmatrelvir) and HIV-1 protease (ritonavir) optimized by the strong CYP3A inhibitory activity of ritonavir (Akinosoglou et al., 2022).

### 5.1 Limitations of the study

A limitation of this study is that a limited dose span and restricted dose escalations were used, which did not allow for the exploration of the full range of effects of the drugs. However, the restrictions were important in the presence of usual budget constraints, and there is a need to focus on achievable concentrations in the human plasma because off-label use rather than expanded label was the theoretical framework of this study. Such full-range dose studies will still need to be done if expanded drug labeling involving alternative delivery systems is considered. Another limitation is that the studies were conducted between three different collaborating laboratories that may have different laboratory fidelities. Nonetheless, this was a pre-identified challenge, and steps were taken to harmonize workflow between laboratories and thus enhance data integrity. Even then, no experiment conducted in one laboratory was re-run in another collaborating laboratory. Also, although we estimated IC_50_ in this study (**Supplementary 1**), the point estimates should be interpreted with caution because of the possibility of non-monotonicity in the response, a known drawback of IC_50_/EC_50_ estimations (Vandenberg et al., 2012) (for example, the CPE and M^PRO^ inhibitions of retapamulin appears “n” shaped and “u” shaped, respectively) (**Figures 1**, **3**). The 95% CI estimates of the IC_50_ provide some reassurance, but again, these were estimated by using the accepted method of inputting unknown data from standard curves (Prichard and Barwick, 2003; Nusholtz et al., 2010). Nonetheless, the strengths of the study are the use of validated cell-based assays, ease of running the experiments in moderately equipped laboratories (International Standard Organization Level 2 (Schneider et al., 2017), and the detection of an acceptable level of effects at therapeutic concentrations of the drugs.

## 6 Conclusion

In this study, we identified erythromycin, retapamulin, pyridoxine, and folic acid as potential therapeutic agents for COVID-19 and provided evidence that ivermectin may not be effective. Because full or close to full effect (100% inhibition) is an ideal target of drug therapy and none of the drugs achieved this at therapeutic doses, combination therapy is recommended though synergism may not be guaranteed. Such combinations may be evaluated in a randomized controlled trial using the basket or umbrella design (Janiaud et al., 2019). Such a trial will need careful design and funding consideration. Meanwhile, we consider that the evidence provided by this study is sufficient for consideration of off-label use of these drugs in COVID-19 situations, given that the evidence is consistent and comparable with those available for drugs currently in clinical trials (Chavda et al., 2022; Kim, 2022) and probably superior to those currently on off-label use (Shojaei and Salari, 2020; Dasgupta, 2021). We also recommend that all off-label prescriptions, while maintaining standard ethical requirements, should include robust and objective documentation of patients’ status and drug dosing to provide *preliminary insight* into the effectiveness of the drugs. Such *preliminary insight documentation* should be routine in off-label prescriptions but may not and cannot replace randomized trials. In this regard, the risk of abuse of documented off-label prescription, like using it as a convenient alternative to clinical trials, should be recognized and mitigated (Dasgupta, 2021).

## Data availability statement

The datasets presented in this study can be found in online repositories. The names of the repository/repositories and accession number(s) can be found below: https://www.dropbox.com/scl/fo/puu4ern2b1y4xnlhosni5/h?rlkey=px8y36a9724us9pgfne2bgtzf&dl=0.

## Author contributions

SB: Validation, Visualization, Conceptualization, Formal analysis, Funding acquisition, Methodology, Project administration, Software, Supervision, Writing – original draft, Writing – review & editing, Data curation, Resources. MI: Supervision, Validation, Writing – review & editing, Formal analysis, Funding acquisition, Methodology. MB: Data curation, Funding acquisition, Methodology, Supervision, Writing – review & editing. AY: Conceptualization, Supervision, Visualization, Data curation, Funding acquisition, Investigation, Methodology, Resources, Software, Writing – review & editing. AA: Data curation, Funding acquisition, Investigation, Methodology, Formal analysis, Resources, Software, Writing – review & editing. AS: Conceptualization, Resources, Software, Writing – review & editing, Data curation, Investigation, Methodology, Validation, Visualization. EI: Conceptualization, Formal analysis, Resources, Software, Writing – review & editing. ZH: Conceptualization, Project administration, Resources, Supervision, Visualization, Writing – review & editing. MP: Conceptualization, Resources, Writing – review & editing, Funding acquisition, Software. CO: Conceptualization, Project administration, Resources, Writing – review & editing, Supervision. AYB: Conceptualization, Methodology, Resources, Writing – review & editing, Data curation, Funding acquisition, Investigation, Project administration. YD: Conceptualization, Resources, Writing – review & editing, Methodology, Visualization. IO: Conceptualization, Resources, Writing – review & editing, Supervision.
