# Supplementary material for: Erythromycin, retapamulin, pyridoxine, folic acid, and ivermectin inhibit cytopathic effect, papain-like protease, and MPRO enzymes of SARS-CoV-2
Source: Front Cell Infect Microbiol. 2023 Nov 27;13:1273982. doi: 10.3389/fcimb.2023.1273982 (PMC10711598; doi:10.3389/fcimb.2023.1273982)
Supplement: Supplementary file 1 [file DataSheet_1.pdf]

Supplementary 1

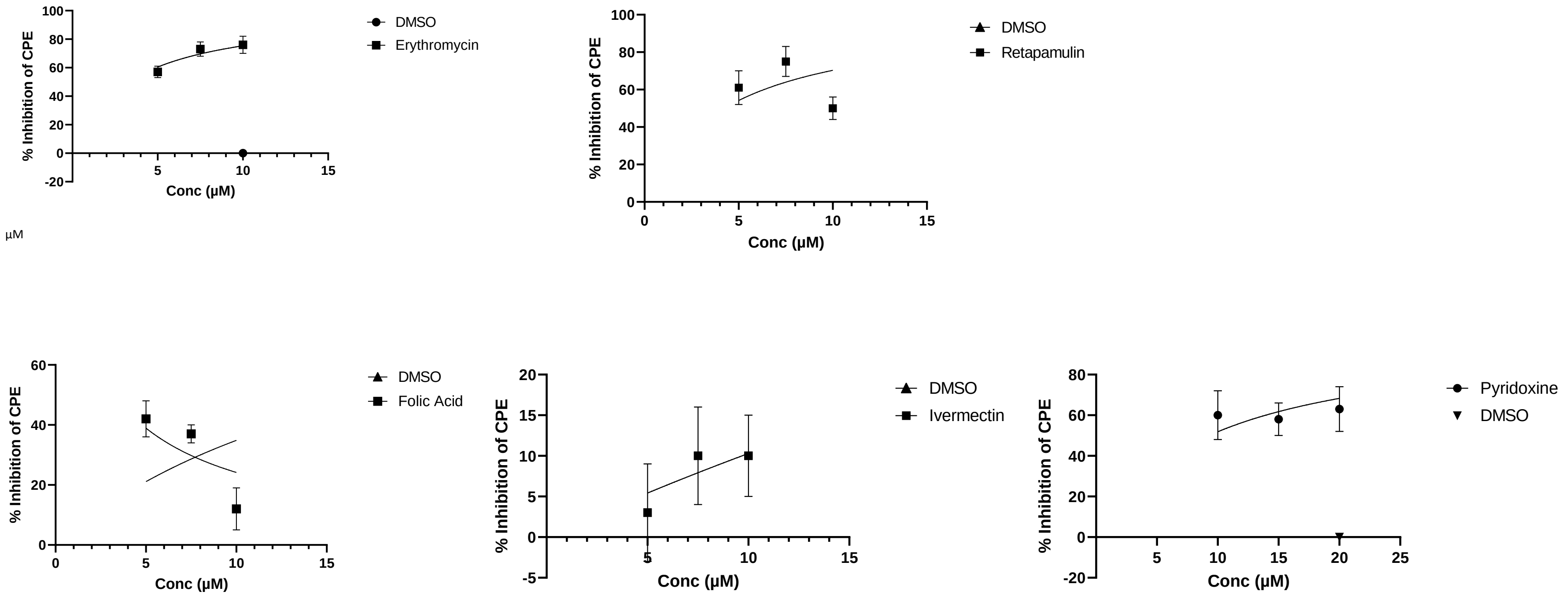

***Estimation of IC50 of inhibition of CPE**.

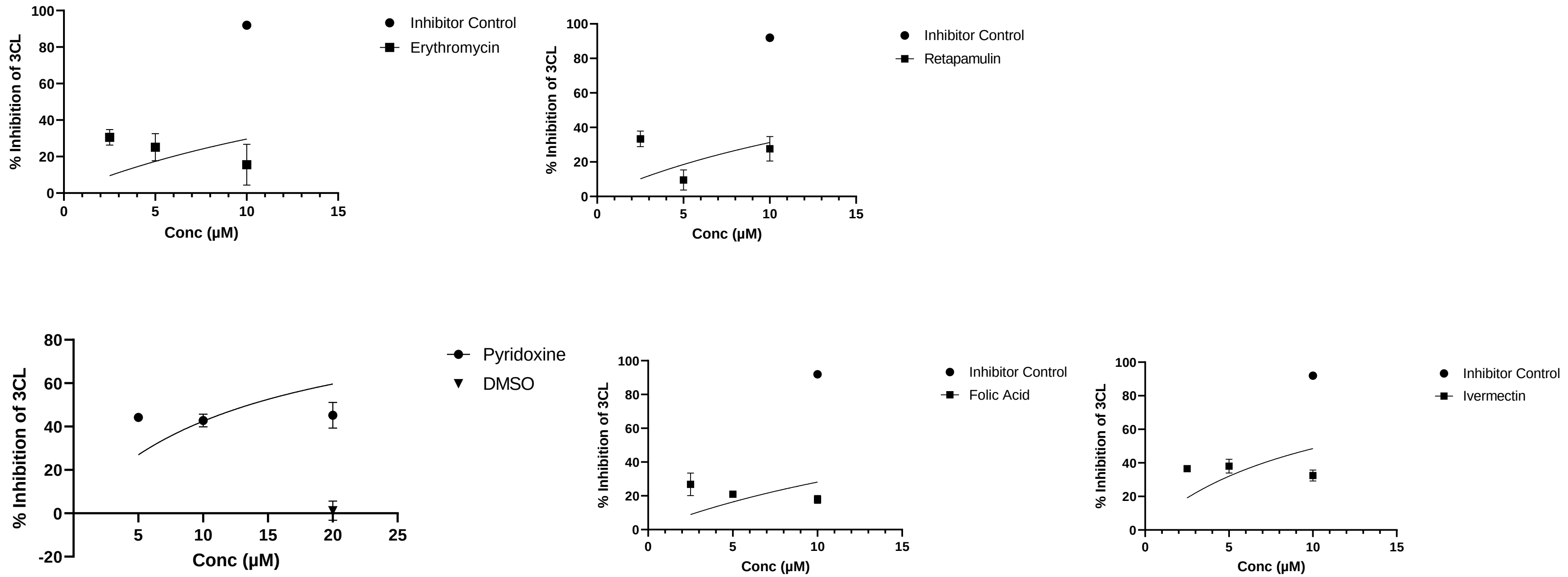

***Estimation of IC50 of inhibition of 3CL**

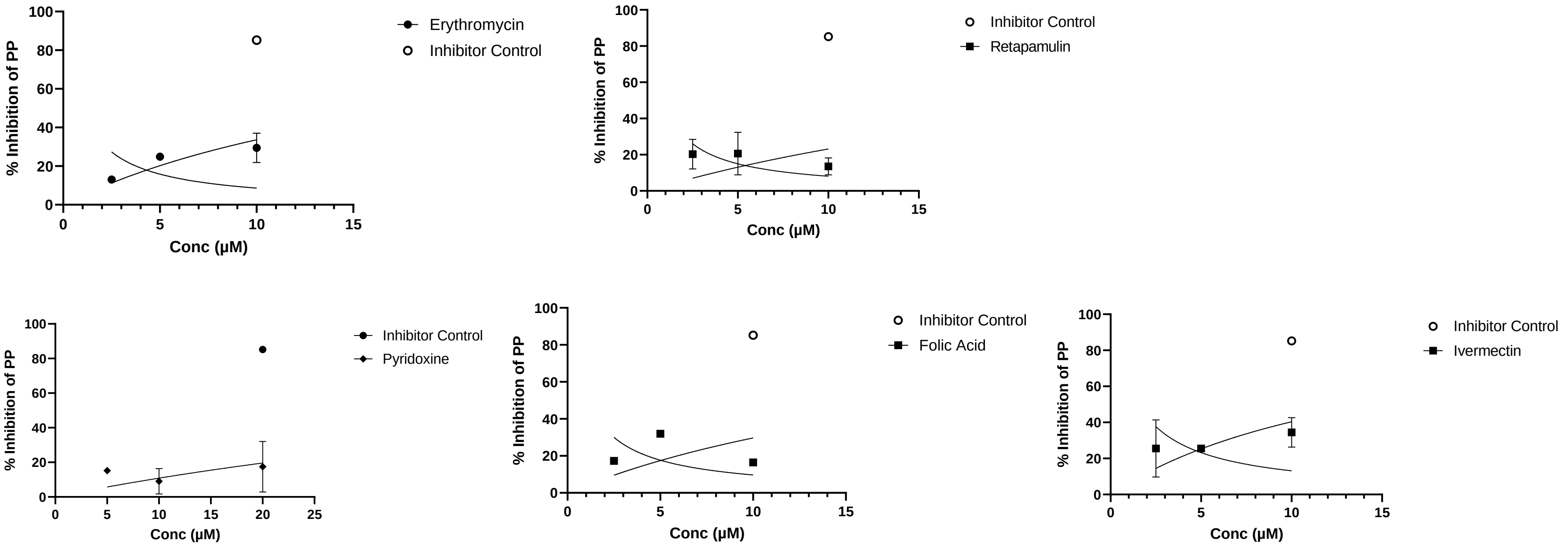

*

***Estimation of IC50 of Inhibition of PP**

*To estimate the IC50, nonlinear three parameter model was executed on GraphPad using least square regression without weighing and with no special handling of outliers. Maximum and minimum responses were constrained to 100% inhibition and 0% inhibition respectively, because these were the biologically plausible limits. Except for Ivermectin, concentrations tested were constrained to those achievable in human plasma at routine recommended doses of the drugs. Concentrations of Ivermectin were restricted to those that have been shown to be effective in previous studies[14-16]. Ninety-five percent (95%) asymmetric (profile-likelihood) Confidence interval was chosen. All enzymes and inhibitor controls were manufacturers' standards.
